# Efficacy results from a 12-month double-blind randomized trial of arimoclomol for treatment of Niemann-Pick disease type C (NPC): Presenting a rescored 4-domain NPC Clinical Severity Scale

**DOI:** 10.1016/j.ymgmr.2025.101233

**Published:** 2025-05-28

**Authors:** Eugen Mengel, Marc C. Patterson, Rosalia M. Da Riol, Mireia Del Toro, Federica Deodato, Matthias Gautschi, Stephanie Grunewald, Sabine Weller Grønborg, Paul Harmatz, Julia B. Hennermann, Bénédicte Héron, Esther M. Maier, Agathe Roubertie, Saikat Santra, Anna Tylki-Szymanska, Lisa LaGorio, Elizabeth Berry-Kravis, Forbes D. Porter, Beth Solomon, Louise Himmelstrup, Travis Mickle, Sven Guenther, Christine í Dali

**Affiliations:** a SphinCS GmbH, Institute of Clinical Science for LSD, Hochheim, Germany; b Departments of Neurology, Pediatrics and Medical Genetics, Mayo Clinic, Rochester, MN, USA; c Regional Coordination Center for Rare Diseases, Academic Hospital ‘Santa Maria della Misericordia’, Udine, Italy; d Pediatric Neurology Department, Vall d'Hebron University Hospital, Barcelona, Spain; e Division of Metabolic Disease and Hepatology, Ospedale Pediatrico Bambino Gesù’, IRCCS, Rome, Italy; f Department of Paediatrics, Division of Endocrinology, Diabetology and Metabolism, and Institute of Clinical Chemistry, Inselspital, University Hospital Bern, Bern, Switzerland; g Department of Metabolic Medicine, Great Ormond Street Hospital for Children NHS Foundation Trust, NIHR Biomedical Research Centre, London, UK; h Center for Inherited Metabolic Diseases, Department of Pediatrics and Adolescent Medicine and Department of Clinical Genetics, Copenhagen University Hospital Rigshospitalet, Copenhagen, Denmark; i Gastroenterology and Nutrition, University of California, San Francisco Benioff Children's Hospital Oakland, Oakland, CA, USA; j University Medical Center Mainz, Center for Pediatric and Adolescent Medicine, Villa Metabolica, Mainz, Germany; k Sorbonne University, Department of Pediatric Neurology –Development Pathology, Reference Center for Lysosomal Diseases, University Hospital Armand Trousseau, AP-HP.SU, FHU I2D2 Paris, France; l Department of Inborn Errors of Metabolism, University of Munich Children's Hospital, Munich, Germany; m Department of Neuropediatrics, Centre Hospitalier Universitaire de Montpellier, INM, INSERM U 1283, Montpellier, France; n Department of Inherited Metabolic Disorders, Birmingham Children's Hospital, Birmingham, UK; o Department of Paediatrics, Nutrition and Metabolic Diseases, The Children's Memorial Institute, Warsaw, Poland; p Department of Communication Sciences and Disorders, College of Health Sciences, Rush University, Chicago, IL, USA; q Departments of Pediatrics, Neurological Sciences, Anatomy and Cell Biology and the RUSH Pediatric Neurosciences F.A.S.T. Center for Translational Research, Rush University Medical Center, Chicago, IL, USA; r *Eunice Kennedy Shriver* National Institute of Child Health and Human Development, National Institutes of Health, Bethesda, MD, USA; s Rehabilitation Medicine Department, Speech Language Pathology Section, Warren G, Magnuson Clinical Research Center, National Institutes of Health, Bethesda, MD, USA; t Zevra Therapeutics, Copenhagen, Denmark; u Zevra Therapeutics, Celebration, FL, USA

**Keywords:** Arimoclomol, Clinical trial, Niemann-Pick disease type C, NPC Clinical Severity Scale, Validity

## Abstract

**Background:**

In the 12-month, randomized, double-blind, placebo-controlled Phase 2/3 NPC-002 study (NCT02612129), arimoclomol significantly reduced annual disease progression versus placebo, measured by the 5-domain NPC Clinical Severity Scale (5DNPCCSS). Arimoclomol has been approved in the US for treatment of Niemann-Pick disease type C (NPC) in combination with miglustat. This paper introduces the rescored 4-domain NPCCSS (R4DNPCCSS) as a *post-hoc* primary endpoint in NPC-002, discusses its validation, and presents the results of the *post-hoc* primary analysis.

**Methods:**

To more accurately assess changes in disease course over a 12-month time period in a heterogeneous group of patients, the Cognition domain was removed from the 5DNPCCSS and the Swallow domain was rescored to reflect linearity in disease progression. Rescoring of the Swallow domain was based on input from clinical NPC and swallow experts from a qualitative interview-based study (*N* = 12), resulting in the R4DNPCCSS. To supplement prior validation analyses, data supporting the overall validity and reliability of the R4DNPCCSS was gathered through additional analyses of construct and convergent validity. The NPC-002 prespecified primary efficacy endpoint analysis based on the 5DNPCCSS score change from baseline to 12 months was repeated with R4DNPCCSS.

**Results:**

Construct validity analysis demonstrated high agreement between the R4DNPCCSS domain scores and the Clinical Global Impression Scale of Severity (CGI-S) and NPC Clinical Database (NPC-cdb) scores. Convergent validity was confirmed by strong correlations between the R4DNPCCSS domains and corresponding items on the Scale for Assessment and Rating of Ataxia (SARA), 9-hole peg test (9-HPT), and Video Fluoroscopic Swallowing Study (VFSS) performance tests. The NPC-002 *post-hoc* primary analysis showed a mean standard error (SE) change in R4DNPCCSS score of 0.35 (0.40) with arimoclomol (*N* = 34) versus 2.05 (0.54) with placebo (*N* = 16), and a treatment effect in favor of arimoclomol over placebo of −1.70 (*p* = 0.0155). In the miglustat subgroup analysis, mean (SE) change in R4DNPCCSS score was −0.23 (1.02) with arimoclomol (*N* = 22) versus 1.92 (3.37) with placebo (N = 12), representing a treatment effect of −2.21 (*p* = 0.0077).

**Conclusion:**

The R4DNPCCSS is a valid and reliable measure of disease progression demonstrating consistent outcomes with the prespecified 5DNPCCSS endpoint. Arimoclomol significantly slowed disease progression through 12 months as measured by the R4DNPCCSS versus placebo.

## 1 Introduction

Niemann-Pick disease type C (NPC) is an ultra-rare disease caused by autosomal recessive pathogenic variants in *NPC1* (∼95 % of cases) or *NPC2*, encoding lysosomal/ endosomal proteins involved in intracellular lipid transport and homeostasis. Mutations in these genes result in defective endosomal-lysosomal cholesterol trafficking, accumulation of multiple lipid species, and impaired lysosomal calcium homeostasis [[1], [2], [3], [4]], which in turn lead to progressive neurodegeneration and premature death [[4], [5], [6], [7], [8]]. The core symptoms of NPC are cerebellar ataxia, dysarthria, dysphagia, progressive dementia and vertical supranuclear gaze palsy [[3], [4], [5],^7^,^9^,^10^]. Disease progression is highly variable. Early onset in neonates and infants is typically associated with rapid deterioration and early mortality. Patients with onset at an older age typically have slower disease progression and may survive into their sixth or seventh decade [5,9]. While disease progression is apparently linear, individuals may show considerable changes in progression rate over time [11,12].

Recently, arimoclomol (MIPLYFFA™, Zevra Therapeutics), an orally bioavailable small molecule crossing the blood-brain barrier, received first approval in the US for the treatment of neurological manifestations of NPC in adults and children aged ≥2 years [13]. The Food and Drug Administration (FDA) approved arimoclomol for use in combination with miglustat, the standard of care for NPC [4]. Miglustat, which inhibits the enzyme glucosylceramide synthase, has been approved for treatment of NPC in Europe and several countries outside the US for over a decade. In the US approximately 70 % of NPC patients currently receive this treatment [14,15]. Clinical trials with miglustat have shown a modest reduction in NPC disease progression [[16], [17], [18]]; long-term follow-up data has shown an impact on disease progression and survival [[19], [20], [21]].

The pivotal Phase 2/3 CT-ORZY-NPC-002 trial (further referred to as the NPC-002 trial) of arimoclomol in NPC (ClinicalTrials.gov identifier: NCT02612129) demonstrated a statistically significant and clinically meaningful reduction in annual disease progression relative to placebo [22]. The primary endpoint of the study was disease progression, assessed using the disease-specific 5-domain NPC Clinical Severity Scale (5DNPCCSS) and analyzed with a mixed model for repeated measures (MMRM), as prespecified in the study protocol [22,23]. The treatment effect of arimoclomol was found to be greater in prespecified subgroups of patients receiving concomitant miglustat and patients aged ≥4 years at treatment initiation [22]. Treatment was well-tolerated.

The 5DNPCCSS that was used as the primary outcome in the NPC-002 study is an abbreviated version of the 17-domain NPCCSS, a disease-specific, clinician-reported clinical severity scale designed to characterize and quantify disease progression in NPC. The NPCCSS has been widely used in NPC clinical care worldwide for over 15 years, significantly contributing to the understanding of the complex, progressive symptoms of NPC [24]. The 5DNPCCSS is validated as an endpoint to measure changes in key domains for NPC trials [23]. The five domains of the 5DNPCCSS were those previously determined to be most clinically relevant to patients, caregivers, and clinicians: Ambulation, Swallow, Cognition, Speech, and Fine Motor Skills [23]. The current paper discusses the rationale behind the introduction of a rescored 4-domain NPCCSS (R4DNPCCSS) as a *post-hoc* primary endpoint in NPC-002, discusses its validity, and presents the results of the *post-hoc* primary analysis.

## 2 Methods

### 2.1 NPC-002 trial

The study design and results of the Phase 2/3 international multi-center NPC-002 trial were previously described in detail by Mengel et al. [22]. The study included a 12-month, randomized, double-blind (DB), placebo-controlled phase followed by a single-arm, 48-month, open-label extension phase. Eligible patients were male or female, aged 2–18 years with a genetically confirmed diagnosis of NPC and either positive filipin staining or elevated cholestane-triol level (>2 × upper limit of normal), at least one neurological symptom, able to walk independently or with assistance and, if treated with miglustat, on a stable dose for at least 6 months. Exclusion criteria included severe liver or renal insufficiency, being neurologically asymptomatic, or having severe uncontrolled epileptic seizures. Patients were randomized 2:1 to arimoclomol or placebo in addition to their routine clinical care [22].

The trial was conducted in accordance with the protocol and in line with the International Council Tripartite Guideline for Harmonisation of Good Clinical Practice (June 1996), the Ethical principles of the Declaration of Helsinki, and local regulatory and legal requirements (International Conference on Harmonisation of Technical Requirements for Registration of Pharmaceuticals for Human Use; World Medical Association). Approval of the study protocol was obtained from the relevant Institutional Review Board/Independent Ethics Committee. Written informed consent was obtained from the participants or their legal guardians.

### 2.2 Introduction of the R4DNPCCSS

#### 2.2.1 Rationale

The prespecified primary efficacy endpoint of the DB phase of NPC-002 was change in disease severity on the 5DNPCCSS from baseline to 12 months [23]. The 5DNPCCSS is an abbreviated version of the 17-domain NPCCSS, comprising five domains: Ambulation, Swallow, Cognition, Speech, and Fine Motor Skills [23].

To more accurately assess changes in disease state over a 12-month time-period in a heterogeneous group of patients and to align with regulatory guidance, the primary outcome measure of the DB phase of trial NPC-002 was supplemented with the R4DNPCCSS endpoint following an evaluation of the 5DNPCCSS scale, which raised potential issues: as for all cognitive tests, the cognition domain ratings rely on the patient environment (e.g., access to services) and may not be sensitive to change within the 12-month trial; furthermore, it was found that the scoring algorithm for the Swallow domain could be improved to provide a more linear interpretation of the scoring categories.

#### 2.2.2 Development and validity of the R4DNPCCSS

To address the issues outlined above, the Cognition domain was removed from the 5DNPCCSS, and input from NPC clinical and swallow experts collected in a qualitative study was used to develop an improved scoring algorithm for the Swallow domain. The qualitative study was carried out by an independent research organization utilizing semi-structured interviews that were designed to gather expert insights on the assessment methods and structure of the Swallow domain. The interviews were followed by cognitive debriefing of the experts on the interview outcomes to inform appropriate rescoring of the NPCCSS Swallow domain and to assess whether the response categories adequately capture severity and progression of swallow dysfunction in the setting of a clinical trial.

To supplement previous validation analyses for the 5DNPCCSS, which are also applicable to the R4DNPCCSS, additional analyses were conducted to further support the construct and convergent validity of the individual domains of the R4DNPCCSS. The construct validity analysis examined whether the severity of each R4DNPCCSS domain score accurately reflects overall disease severity, as measured by two other assessments in NPC-002: the Clinical Global Impression Scale of Severity (CGI-S), a 7-point Likert scale, and the NPC Clinical Database (NPC-cdb) score [11,25], a disease-specific tool covering 72 signs and symptoms of NPC. In this analysis, data from both the DB and open-label phase (active and placebo group combined) of NPC-002 were used. To assess convergent validity, correlations were calculated and distribution patterns were compared between the individual NPCCSS Ambulation, Fine Motor Skills, and Speech domains and subitems of the Scale for Assessment and Rating of Ataxia (SARA). The SARA comprises eight domains, of which the following five assess NPC-relevant symptomatology: Gait, Speech disturbance, Finger chase, Nose-finger test, and Fast alternating hand movements. Additionally, correlations were calculated between the NPCCSS Fine motor skills domain and the 9-hole peg test (9-HPT). Both polychoric and Spearman correlations were used based on data collected at baseline, month 6, and month 12 in the NPC-002 study. Since no functional swallow test was included in the NPC-002 study, convergent validity for the NPCCSS Swallow domain was established using data from the National Institutes of Health (NIH) NPC natural history cohort [8], which applied similar NPCCSS Swallow scoring procedures. This cohort study also included two additional swallow scales: the American Speech-Language-Hearing Association National Outcomes Measurement System (ASHA-NOMS), a 7-point scale assessing swallowing safety, and the Penetration-Aspiration Scale (PAS) [26], which evaluates the risk of penetration or aspiration. Both scales are part of the Video Fluoroscopic Swallowing Study (VFSS) and are broadly used across patient populations with different disorders. To align assessments across subjects in the NIH dataset, visits were grouped into yearly intervals from baseline, and only the first observation per subject per interval was included. Analyses were limited to data from the first 5 years due to low sample sizes in later intervals.

### 2.3 NPC-002 primary and subgroup analyses using R4DNPCCSS

The prespecified primary efficacy endpoint analysis of the NPC-002 study data was based on the 5DNPCCSS score change from baseline to 12 months using a MMRM with a hypothetical estimand [22]. This analysis was repeated with the *post-hoc* primary endpoint R4DNPCCSS using the same source data. The model included treatment, visit, treatment-by-visit interaction, and use of miglustat at baseline as fixed effects and baseline R4DNPCCSS value as a covariate.

For a subgroup analysis in patients receiving miglustat at enrollment, data were analyzed using a treatment-policy estimand that included imputation rules for discontinued patients. All available patient data were used to evaluate the treatment effect. The data for discontinued patients was combined with observed patient data to create a total of 1000 datasets. The treatment difference for each dataset was estimated based on the R4DNPCCSS score change from baseline at month 12 using an analysis of covariance (ANCOVA) with treatment as fixed effect and baseline R4DNPCCSS score as covariate. The results of all datasets were combined using Rubin's rule.

## 3 Results

### 3.1 Rescoring of the Swallow domain

Based on input received from the qualitative study involving eight clinical NPC experts (four from NPC-002, four independent of the study) and four swallow experts, the scoring algorithm of the NPCCSS Swallow domain was optimized to better reflect linearity of dysfunction, without altering the scoring categories (i.e., descriptions of different domain severity levels) (Supplementary file Table S1). The revised scoring reranked dysphagia by frequency (intermittent = 2 or consistent = 3), and assigned a supplemental tube-feeding score of 4, and a tube-feeding-only score of 5. Since the Cognition domain was also removed from the 5DNPCCSS to address the concern that cognition relies on the patient environment and may not be sensitive enough to change over 12 months, the resulting R4DNPCCSS comprises the four domains of Ambulation, Fine motor skills, Speech, and Swallow (Table 1). Individual R4DNPCCSS domain scores range from 0 to 5, based on defined criteria, with higher scores representing more severe clinical impairment. The total score range is 0 to 20 points.

**Table 1:** Definitions and scoring for each domain of the R4DNPCCSS.

Domain score	Ambulation	Fine Motor Skills	Speech	Swallow
0	Normal	Normal	Normal	Normal
1	Clumsy, bangs into things	Slight dysmetria/dystonia (independent manipulation)	Mild dysarthria (easily understood)	Cough while eating
2	Ataxic unassisted gait	Mild dysmetria/dystonia (requires little to no assistance, able to feed self easily)	Severe dysarthria (difficult to understand)	Intermittent dysphagia
3	–	–	Non-verbal/functional communication skills for needs	Dysphagia
4	Assisted ambulation	Moderate dysmetria/dystonia (limited fine motor skills; difficulty feeding self	–	Nasogastric tube or gastric tube for supplemental feeding
5	Wheelchair dependent	Severe dysmetria/dystonia (gross motor limitation, requires assistance for self-care activities)	Minimal communication	Nasogastric tube or gastric tube feeding only

R4DNPCCSS: rescored 4-domain Niemann-Pick disease type C Clinical Severity Scale.

See Supplementary file Table S1 for more details on how items in the Swallow domain are scored.

### 3.2 Validity of the R4DNPCCSS

#### 3.2.1 Applicable data from the 5DNPCCSS validation

Most of the validation work was initially conducted using all five domains of the 5DNPCCSS, prior to revisiting the scoring methodology for the Swallow domain and removing the Cognition domain, as previously reported [23]. Briefly, these analyses demonstrated strong correlations between the 5DNPCCSS and the 17-item NPCCSS total score (excluding the Auditory brainstem response and Hearing domains) (r^2^ = 0.97). Additionally, convergent validity of the 5DNPCCSS total score and the Fine motor skills domain score against the 9-HPT (both r^2^ = 0.65) and between the 5DNPCCSS total score and SARA (r^2^ = 0.86) was demonstrated. The results of these analyses also apply to the R4DNPCCSS since scoring of the Ambulation, Speech, and Fine motor skills domains were not changed and descriptions of the Swallow response categories were maintained.

#### 3.2.2 NPC and swallow expert feedback on the R4DNPCCSS

The qualitative study involving clinical NPC and swallow experts provided additional evidence for the validity and standardization of the Swallow domain. Details on the clinical background and experience of the experts are provided in Table S2 and Table S3 of the Supplementary file. Overall, the experts agreed that the response categories of the Swallow domain capture relevant clinical features in proper order of severity and can track changes over time across ages, thus allowing for consistent patient assessments in NPC-002. They noted that the scoring method effectively reflects progression in disease severity, with each stepwise increase in dysfunction corresponding to a numeric score increase. However, multiple experts indicated that a linear rather than an additive scoring system would be more appropriate to assess swallow function.

#### 3.2.3 Construct validity of the R4DNPCCSS

Construct validity analyses of the individual domains of the R4DNPCCSS demonstrated high agreement between each of the R4DNPCCSS scores and the NPC-cdb score and CGI-S. A higher score of each of the individual R4DNPCCSS domains corresponded to a higher adjusted mean estimate of disease severity on both the CGI-S and NPC-cdb, which strongly supports the construct validity of each of the four domains (Supplementary file, Fig. S1).

#### 3.2.4 Convergent validity of the R4DNPCCSS

Additional evidence for convergent validity of the NPCCSS Ambulation, Fine motor skills, and Speech domains was provided by correlation analyses showing high agreement between the domains of the R4DNPCCSS and the performance-based instruments that were employed in the NPC-002 study. Overall, polychoric (r_pc_) and Spearman (r_S_) correlations between these NPCCSS domains and corresponding items on various performance-based tests were found to be moderate (0.40–0.60) to strong (≥0.60) (Table 2) [27].

**Table 2:** Convergent validity analysis: correlations between absolute scores of NPCCSS Ambulation, Fine motor skills and Speech and related items of other performance tests (SARA and 9-HPT) at baseline and months 6 and 12 of the NPC-002 study (*N* = 50).

NPCCSS domain (score range)	Performance test item	n at 0, 6 and 12 months	Polychoric correlation at 0, 6 and 12 months			Spearman correlation at 0, 6 and 12 months		
			0	6	12	0	6	12
Ambulation (0–5, score 3 is not an option)	SARA Gait (0–8)^a^	49, 44, 41	0.91	0.97	0.94	0.85	0.92	0.90
Fine motor skills (0–5, score 3 is not an option)	SARA Finger chase (0–4)^a^	47, 43, 40	0.74	0.85	0.93	0.66	0.76	0.85
	SARA Nose-finger test (0–4)^a^	47, 43, 40	0.71	0.85	0.88	0.62	0.76	0.81
	SARA Fast alternating hand movements (0–4)^a^	46, 43, 40	0.67	0.82	0.82	0.58	0.73	0.76
	9-HPT (seconds)^b^	31, 26, 25	0.45	0.73	0.72	0.58	0.84	0.77
Speech (0–5, score 4 is not an option)	SARA Speech disturbance^a^	49, 44, 41	0.94	0.99	0.97	0.89	0.94	0.92

9-HPT: 9-hole peg test; N: number of subjects in population; n: number of observations; NPCCSS: Niemann-Pick disease type C Clinical Severity Scale; SARA: Scale for the Assessment of Rating of Ataxia.

a Normal cerebellar function = 0, unable to perform the test = highest score.

b Completion of the 9-HPT included one practice test for both the dominant and non-dominant hand without timing to familiarize the patient with the test followed by one timed test for each hand.

Strong polychoric and Spearman correlations were seen at baseline and at months 6 and 12 between the NPCCSS Ambulation domain and the SARA Gait item and between the NPCCSS Fine motor skills domain and the SARA Finger chase, Nose-finger test and Fast alternating hand movements items. The NPCCSS Fine motor skills score was also correlated with the performance test 9-HPT showing moderate to strong polychoric correlations at baseline and months 6 and 12. As a considerable number of patients in the trial could not complete the 9-HPT (38–43 % per visit), the number of patients with data for this comparison was lower than for the SARA scale items. Strong correlations were also found between NPCCSS Speech and SARA Speech disturbance at all time points. The histograms for score patterns at baseline, and months 6 and 12 showed generally similar distribution patterns for all scores that were compared, supporting that the observed strong correlations are statistically meaningful. Overall, these results support the convergent validity of the NPCCSS Ambulation, Fine Motor Skills and Speech domains.

The convergent analyses of the NPCCSS Swallow domain, using data from the NIH natural history study (clinicaltrials.gov NCT00344331), showed moderate to strong correlations with the ASHA-NOMS and PAS for both absolute values and changes from baseline (Table 3). Additionally, mean scores for NPCCSS swallow, ASHA-NOMS, and PAS in the NIH natural history cohort over a 10-year period showed very similar patterns between the three scales. Score distributions were similar between all three instruments. Together, these findings support that the NPCCSS Swallow domain truly reflects progression in Swallow dysfunction and track changes in swallowing dysfunction over time.

**Table 3:** Correlations between absolute values and change scores of NPCCSS Swallow and ASHA-NOMS and PAS; all visits from baseline to year 5 (natural history cohort, *N* = 120).

Correlation	NPCCSS Swallow vs ASHA-NOMS		NPCCSS Swallow vs PAS	
	Absolute (*n* = 252)	Changes (*n* = 132)	Absolute (*n* = 251)	Changes (*n* = 131)
Polychoric	0.81	0.65	0.82	0.56
Spearman	0.59	0.53	0.57	0.47

ASHA-NOMS: American Speech-Language-Hearing Association National Outcomes Measurement System; N: number of subjects in the cohort; n: number of observations; NIH, National Institutes of Health; NPCCSS: Niemann-Pick disease type C Clinical Severity Scale; PAS: Penetration-Aspiration Scale (methods as described by Solomon et al. [26,28]).

### 3.3 NPC-002 primary analysis using R4DNPCCSS

#### 3.3.1 Study population

The R4DNPCCSS was used as a *post-hoc* primary outcome measure in NPC-002. As previously described, of 50 patients who started the DB placebo-controlled phase of this study (arimoclomol *N* = 34; placebo *N* = 16), 42 completed 12 months of treatment [22]. Reasons for withdrawal, baseline demographics and disease characteristics have been published previously [22]. The mean age for the total cohort was 11.1 years. Baseline mean (SD) R4DNPCCSS score was 9.2 (5.8) in the arimoclomol group and 6.7 (5.2) in the placebo group.

#### 3.3.2 Primary analysis

Table 4 summarizes the primary analysis results for both the prespecified endpoint 5DNPCCSS, as previously reported by Mengel et al. [22], and the *post-hoc* R4DNPCCSS endpoint. Mean scores at baseline and 12 months are presented in the supplementary file, Table S4. Both analyses demonstrate that patients treated with arimoclomol experienced significantly slower disease progression compared to those receiving placebo during the DB phase of NPC-002. In the *post-hoc* analysis, the mean (SE) change in R4DNPCCSS score was 0.35 (0.40) in the arimoclomol group versus 2.05 (0.54) in the placebo group, resulting in a statistically significant and clinically meaningful treatment effect in favor of arimoclomol over placebo of −1.70 (*p* = 0.0155).

**Table 4:** Primary efficacy endpoint analysis based on the prespecified 5DNPCCSS endpoint and the *post-hoc* R4DNPCCSS endpoint (NPC-002 study).

Endpoint	Arimoclomol (N = 34)	Placebo (N = 16)	LSM difference (95 % CI)	*p*-value
	LSM (SE)	LSM (SE)		
Change in 5DNPCCSS from baseline to 12 months [22]	0.72 (0.40)	2.11 (0.55)	−1.40 (−2.76, −0.03)	0.0456
Change in R4DNPCCSS from baseline to 12 months	0.35 (0.40)	2.05 (0.54)	−1.70 (−3.05, −0.34)	0.0155

Mixed effects model for repeated measurements analysis including treatment, visit, treatment-by-visit interaction, and use of miglustat at baseline as fixed effects and baseline R4DNPCCSS or 5DNPCCSS value as a covariate.

5DNPCCSS: 5-domain Niemann-Pick disease type C Clinical Severity Scale; CI: confidence interval; LSM: least squares mean; N: number of patients in population; R4DNPCCSS: rescored 4-domain Niemann-Pick disease type C Clinical Severity Scale; SE: standard error.

### 3.4 Subgroup analysis of R4NPCCSS in patients receiving miglustat

**Fig. 1:**
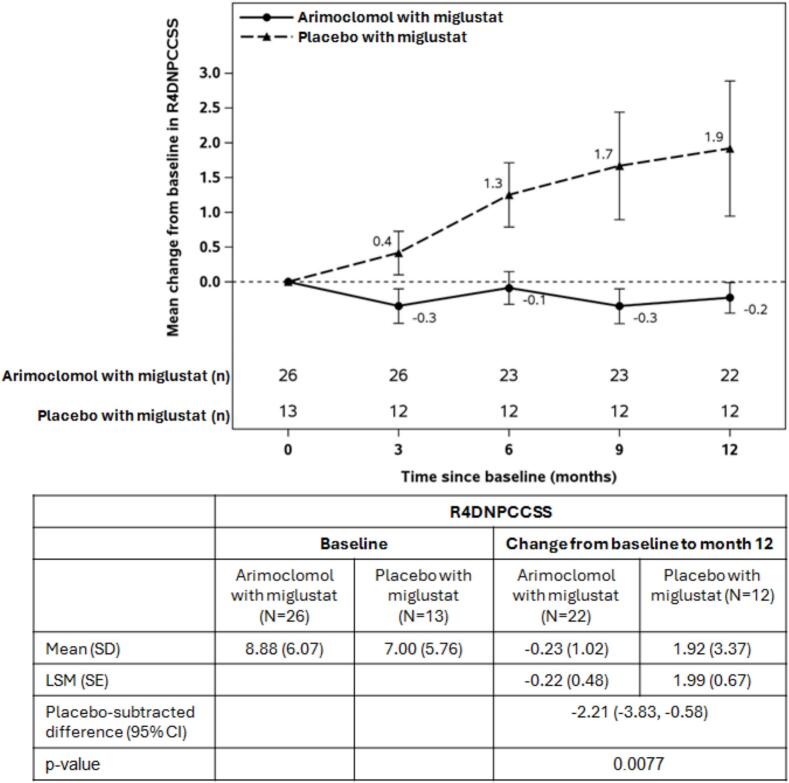
Arimoclomol and placebo change from baseline in R4DNPCCSS over 12 months in the subgroup of patients who also received miglustat (NPC-002 study). Changes in R4DNPCCSS from baseline to month 12 were compared using an analysis of covariance (ANCOVA) model fitted with treatment and baseline R4DNPCCSS as covariate. CI: confidence interval; LSM: least squares mean; N: number of patients in population; R4DNPCCSS: rescored Niemann-Pick disease type C Clinical Severity Scale; SE: standard error.

Thirty-nine (78 %) subjects in the NPC-002 study, equally distributed over the arimoclomol and placebo groups, used concomitant miglustat at enrollment as part of routine clinical care. A subgroup analysis in these patients showed a mean (SE) change in R4DNPCCSS score of −0.23 (1.02) in the arimoclomol group versus 1.92 (3.37) for placebo, with a statistically significant and clinically meaningful treatment effect in favor of arimoclomol over placebo of −2.21 points (*p* = 0.0077) (Fig. 1). Data for the subgroup of patients who did not take miglustat are not presented given the small sample size (three in the placebo arm and eight in the arimoclomol arm) that resulted in marked baseline imbalances between the treatment arms preventing generalizability and reliability of inferences from any statistical analysis.

## 4 Discussion

The primary analysis of the 12-month DB randomized trial of arimoclomol (NPC-002) previously demonstrated a significant and clinically meaningful impact of arimoclomol on disease progression over 12 months compared to placebo [22]. The data presented here confirm that these findings are reproducible using the R4DNPCCSS, introduced as a *post-hoc* primary endpoint in NPC-002 to more accurately assess changes in disease state over a 12-month time period in a heterogeneous group of patients and to align with regulatory guidance. This revised R4DNPCCSS endpoint was adapted from the original 5DNPCCSS by omitting the Cognition domain and simplifying the scoring algorithm for the Swallow domain to improve the linearity of response categories with disease severity. Notably, while the R4DNPCCSS offers better alignment with disease severity, it will be more challenging to compare with natural history studies which used the original score. The Cognition domain was removed to address the concern that cognition relies on the patient environment and may not be sensitive enough to short-term changes. While this means the scale no longer directly captures cognitive decline, a recognized marker of NPC progression, the validation analysis shows that it remains a robust and valuable tool for tracking disease progression, with a focus on more reliably measurable domains.

Prior and new validation analyses support the new R4DNPCCSS tool as a valid and reliable measure of disease progression in NPC. Applicable previous validation work completed for the domains of the 5DNPCCSS showed significant (*p* ≤ 0.0001) correlations between the 5DNPCCSS total score versus the SARA total score, the NPCCSS Fine motor skills versus the 9-HPT, and the 5DNPCCSS total score versus the 9-HPT, providing substantial evidence of convergent validity of the 5DNPCCSS [23]. These findings remain relevant for the R4DNPCCSS since scoring of the Ambulation, Speech, and Fine motor skills domains were not changed and descriptions of the Swallow response categories were maintained.

The additional validation analyses presented here show that the individual domains of the R4DNPCCSS are able to capture clinical progression in NPC disease severity, support that experienced and trained clinicians can interpret and differentiate the response options within each domain of the R4DNPCCSS, and support the suitability of the Swallow domain for assessing progression of swallow dysfunction. In addition, construct validity of the Ambulation, Fine motor skills, and Speech domains was confirmed by high agreement between each of the 4DNPCCSS scores and the disease-specific NPC-cdb score as well as global severity of disease as assessed by the CGI-S. Convergent validity of each of the NPCCSS domains was documented by correlations to performance-based measures and distribution graphs showing high agreement between the NPCCSS domains and the performance-based instruments that were used in the NPC-002 and the NIH natural history [8,22]. Since the scales are intended to measure different aspects of the disease and due to differences in individual score ranges and category descriptors, the scores cannot be perfectly mapped between performance tests and related NPCCSS domains. Nevertheless, strong correlations were found that validate all four domains of the R4DNPCCSS and confirm that this tool is well-defined and standardized for consistent use across patients and clinical sites in NPC clinical trials.

In line with the findings from the prespecified primary endpoint (5DNPCCSS) analysis [22], the *post-hoc* primary analysis using the R4DNPCCSS endpoint demonstrated a statistically significant treatment effect favoring arimoclomol over placebo, with a clinically meaningful difference of −1.70 during the 12-month DB phase (*p* = 0.0155), indicating a slowing of disease progression. The pre-specified primary MMRM analysis, yielding a hypothetical estimand, assessed the expected benefit on the 5DNPCCSS that a future population might experience after 12 months of uninterrupted exposure to arimoclomol in addition to routine clinical care, compared to routine clinical care alone. This estimand provides NPC clinicians with a basis to discuss the predicted clinical outcomes of sustained arimoclomol treatment over a year with patients and their caregivers. However, the MMRM analysis excluded data from patients who prematurely discontinued the study or died prior to 12 months. Since a review of the excluded data revealed evidence of disease progression, a different statistical approach was used for the subgroup analysis of miglustat, incorporating imputation rules to account for patients who discontinued. This analysis showed a significantly slower rate of disease progression, as measured by the R4DNPCCSS, in patients receiving both arimoclomol and miglustat compared to those on miglustat alone. The treatment effect was −2.21 in favor of arimoclomol and miglustat (*p* = 0.0077). These findings align with results from the prespecified subgroup analysis using the 5DNPCCSS endpoint, which showed a treatment difference of −2.06 (*p* = 0.006). Of note, the original anchor-based analyses for the validation of the 5DNPCCSS suggested that progressing beyond a 1-point worsening on the 5DNPCCSS would be clinically meaningful and preventing a 2-point worsening would be a viable treatment goal [23]. Other findings from the primary analysis that were previously reported, including secondary endpoints and safety outcomes, remain valid [22].

## 5 Conclusions

Overall, the presented data demonstrate that R4DNPCCSS is a valid and reliable measure of disease progression that is suitable for use across patients and clinical sites in NPC clinical trials. Arimoclomol significantly slowed disease progression through 12 months of treatment, as measured by the R4DNPCCSS, versus placebo in the full analysis set and the miglustat subgroup of the NPC-002 study, confirming the statistically significant and clinically meaningful reduction in disease progression observed with the prespecified 5DNPCCSS endpoint [22].

The following are the supplementary data related to this article.

Supplementary material Table S1. A) The original Swallow domain scoring methodology for the 5DNPCCSS. Scores with + are additive to the category score (cough while eating). B) Updated Swallow domain scoring methodology. The updated scoring reflects distinct categorization with a more linear scoring pattern. A guideline for scoring the rescored Swallow domain is provided below the table
Table S2. Population background data of the qualitative study involving clinical experts and swallow experts
Table S3. Experience of clinical experts and swallow experts involved in the qualitative study with the 5DNPCCSS/NPCCSS
Fig. S1. Severity of individual R4DNPCCSS domains versus global severity of disease assessed by CGI-S (top) and NPC-cdb (bottom) (NPC-002, FAS)
Table S4. 5DNPCCSS and R4DNPCCSS scores at baseline and 12 months of treatment Supplementary material

## CRediT authorship contribution statement

**Eugen Mengel:** Writing – review & editing, Methodology, Investigation, Formal analysis, Data curation, Conceptualization. **Marc C. Patterson:** Writing – review & editing, Methodology, Investigation, Formal analysis, Data curation, Conceptualization. **Rosalia M. Da Riol:** Writing – review & editing, Investigation, Formal analysis, Data curation. **Mireia Del Toro:** Writing – review & editing, Investigation, Formal analysis, Data curation. **Federica Deodato:** Writing – review & editing, Investigation, Formal analysis, Data curation. **Matthias Gautschi:** Writing – review & editing, Investigation, Formal analysis, Data curation. **Stephanie Grunewald:** Writing – review & editing, Investigation, Formal analysis, Data curation. **Sabine Weller Grønborg:** Writing – review & editing, Investigation, Formal analysis, Data curation. **Paul Harmatz:** Writing – review & editing, Investigation, Formal analysis, Data curation. **Julia B. Hennermann:** Writing – review & editing, Investigation, Formal analysis, Data curation. **Bénédicte Héron:** Writing – review & editing, Investigation, Formal analysis, Data curation. **Esther M. Maier:** Writing – review & editing, Investigation, Formal analysis, Data curation. **Agathe Roubertie:** Writing – review & editing, Investigation, Formal analysis, Data curation. **Saikat Santra:** Writing – review & editing, Investigation, Formal analysis, Data curation. **Anna Tylki-Szymanska:** Writing – review & editing, Investigation, Formal analysis, Data curation. **Lisa LaGorio:** Writing – review & editing, Investigation, Formal analysis, Data curation. **Elizabeth Berry-Kravis:** Writing – review & editing, Investigation, Formal analysis, Data curation. **Forbes D. Porter:** Data curation, Formal analysis, Writing – review & editing, Investigation. **Beth Solomon:** Data curation, Formal analysis, Investigation, Writing – review & editing. **Louise Himmelstrup:** Writing – review & editing, Investigation, Formal analysis, Data curation. **Travis Mickle:** Writing – review & editing, Investigation, Formal analysis, Data curation. **Sven Guenther:** Writing – review & editing, Software, Investigation, Formal analysis, Data curation. **Christine í Dali:** Writing – review & editing, Methodology, Investigation, Formal analysis, Data curation, Conceptualization.

## Ethics approval and consent to participate

The trial (ClinicalTrials.gov identifier: NCT02612129) protocol and associated documentation were approved by the relevant independent ethics committees and/or institutional review boards, and written informed consent was obtained at enrollment from either the patient or their legal guardian.

## Funding

The research was funded by Zevra Therapeutics Inc., who were involved in all stages of the trial design, data collection, analysis, and interpretation of the results.

## Declaration of competing interest

Eugen Mengel has received investigator fees and/or consultant honoraria from Cyclo Therapeutics, Amicus, Idorsia, Intrabio, Denali, JCR, Prevail, Freeline Therapeutics, Alexion, Zevra, Sanofi Genzyme, and Takeda.

Marc C. Patterson has received honoraria for advisory boards from Zevra (paid to Mayo Clinic), and research support (paid to Mayo Clinic) from Amicus, Glycomine, Idorsia, Zevra, and Shire-Takeda; he holds stock in IntraBio.

Rosalia *M. Da* Riol has received travel expenses and congress fees reimbursements from Sanofi Genzyme and Takeda.

Mireia Del Toro has received consulting fees and speaker honoraria, travel expenses, and congress fees from Biomarin, Sanofi Genzyme, and Takeda, and is an investigator for industrial trials (Zevra, Takeda, Vtesse-Sucampo-Mallinckrodt).

Federica Deodato has received speaker honoraria from Sanofi Genzyme and Takeda, and travel reimbursement and congress fees from Actelion, Sanofi Genzyme, and Takeda.

Matthias Gautschi has received consulting fees from Sanofi Genzyme, and travel expenses and congress fees from Takeda, and is an investigator for industrial trials from Horizon, Idorsia, Kaleido, Mallinckrodt, Zevra, and Intrabio.

Stephanie Grunewald has received consultancy funding from Hyperion, Moderna, Nutricia, Sobi, Glycomine, and Ultragenyx, and has participated in commercially funded research and received travel grants from Zevra.

Sabine Weller Grønborg has received travel expenses and congress fee reimbursements from Sanofi Genzyme, participated in Orchard Therapeutics advisory board and sponsored meetings, and has received speaker honoraria from Actelion and Novo Nordisk.

Paul Harmatz has provided consulting support to and/or has received grant support from Adrenas, Aeglea, Alexion, Armagen, ASPA, Audentis, Azafros, BioMarin, Bridgebio, Calcilytics, Capsida, Chiesi, Denali, Edigene, Enzyvant, GC Pharma, Genzyme, Grace Science, Homology, Idorsia, Inventiva, JCR, Mirum Pharma, Neurogene, Novel Pharma, Orchard Therapeutics, Paradigm, Pfizer, 10.13039/100013223PTC Therapeutics, QED, Rallybio, RegenXbio, Renoviron, Saliogen, Sangamo, Shire, Sobi, Takeda, and Ultragenyx, and Zevra.

Julia B Hennermann received honoraria and/or travel expenses from Amicus, Chiesi, Immedica, Takeda, and Sanofi.

Bénédicte Héron has received honoraria for advisory boards from Orchard Therapeutics, Actelion, Takeda, and Zevra; received honoraria/travel support from Actelion, BioMarin, Shire/Takeda, 10.13039/100013995Sanofi Genzyme; is principal investigator for Abeona, Zevra, Lysogene, Mallincrodt, Idorsia, JCR Pharmaceuticals, and Chiesi studies; is an expert consultant for Lysogene, Takeda and Zevra.

Esther M. Maier has received fees from Sanofi.

Agathe Roubertie has no conflicts of interest.

Saikat Santra has participated in advisory boards for Moderna, Sobi, Immedica, BioMarin and Ultragenyx, and has participated in commercially funded research and received travel grants from Zevra, Biomarin, Takeda and Sanofi Genzyme.

Anna Tylki-Szymanska has received speaker honoraria and/or travel grants from BioMarin, Chiesi, Sanofi Genzyme, and Takeda.

Lisa LaGorio has received funding from Orphazyme/Zevra for the design and validation of the Swallow domain of the 4NPCCSS, and for consulting for and participating in the FDA AdComm meeting. Dr. LaGorio has also received funding for consulting with the Florida Dysphagia Institute, and honoraria from Aspire Respiratory Products, Jackson University, the Beijing Language and Culture University, and the South Cook County Speech and Hearing Association.

Elizabeth Berry-Kravis has received funding from Acadia, Alcobra, AMO, Asuragen, Avexis, Biogen, BioMarin, Cydan, Engrail, Erydel, Fulcrum, GeneTx, GW, Healx, Ionis, Jaguar, Kisbee, Lumos, Marinus, Mazhi, Moment Bioscience, Neuren, Neurogene, Neurotrope, Novartis, Orphazyme/Kempharm/Zevra, Ovid, PTC Therapeutics, Retrophin, Roche, Seaside Therapeutics, Taysha, Tetra, Ultragenyx, Yamo, Zynerba, and Vtesse/Sucampo/Mallinckrodt Pharmaceuticals, to consult on trial design or run clinical or lab validation trials in genetic neurodevelopmental or neurodegenerative disorders, all of which is directed to RUMC in support of rare disease programs; Dr. Berry-Kravis receives no personal funds and RUMC has no relevant financial interest in any of the commercial entities listed.

Forbes D. Porter receives funding from the Intramural Research Program of the National Institutes of Health. His research program receives funding through Cooperative Research and Development Agreements between the NIH and Vtesse/Sucampo/Mallinckrodt/Mandos Heath, Scenic Biotech, Amicus, Beyond Batten Disease Foundation, and Ultragenyx. Dr. Porter receives no personal funds and has no relevant financial interest in any of the commercial entities listed. Dr. Porter has received funds from multiple patient support organizations related to both Smith-Lemli-Opitz syndrome and Niemann-Pick disease, type C.

Beth Solomon has no conflicts of interest.

Christine í Dali and Louise Himmelstrup are employees and shareholders of Zevra Pharmaceuticals Inc.

Travis Mickle and Sven Guenther are consultants and shareholders of Zevra Pharmaceuticals Inc.

## Data Availability

The trial protocol and Statistical Analysis Plans will become publicly available. Study information will be posted on https://clinicaltrials.gov/ct2/show/NCT02612129. The data that support the findings of this trial are available from Zevra but restrictions apply to the availability of these data, which were used under license for the current trial, and so are not publicly available. Data are however available from the authors upon reasonable request and with permission of Zevra.
