# Supplementary material for: Efficacy results from a 12-month double-blind randomized trial of arimoclomol for treatment of Niemann-Pick disease type C (NPC): Presenting a rescored 4-domain NPC Clinical Severity Scale
Source: Mol Genet Metab Rep. 2025 May 28;43:101233. doi: 10.1016/j.ymgmr.2025.101233 (PMC12166820; doi:10.1016/j.ymgmr.2025.101233)
Supplement: Supplementary material — Table S1. A) The original Swallow domain scoring methodology for the 5DNPCCSS. Scores with + are additive to the category score (cough while eating). B) Updated Swallow domain scoring methodology. The updated scoring reflects distinct categorization with a more linear scoring pattern. A guideline for scoring the rescored Swallow domain is provided below the table Table S2. Population background data of the qualitative study involving clinical experts and swallow experts Table S3. Experience of clinical experts and swallow experts involved in the qualitative study with the 5DNPCCSS/NPCCSS Fig. S1. Severity of individual R4DNPCCSS domains versus global severity of disease assessed by CGI-S (top) and NPC-cdb (bottom) (NPC-002, FAS) Table S4. 5DNPCCSS and R4DNPCCSS scores at baseline and 12 months of treatment [file mmc1.pdf]

Supplementary file

Table S1. A) The original Swallow domain scoring methodology for the 5DNPCCSS. Scores with + are additive to the category score (cough while eating). B) Updated Swallow domain scoring methodology. The updated scoring reflects distinct categorization with a more linear scoring pattern. A guideline for scoring the rescored Swallow domain is provided below the table

A

Original Swallow Domain Scoring Methodology	Score
Normal, no dysphagia	0
Cough while eating	1
Intermittent dysphagia with liquids	+ 1
Intermittent dysphagia with solids	+ 1
Dysphagia with liquids	+ 2
Dysphagia with solids	+ 2
Nasogastric tube or gastric tube for supplemental feeding	4
Nasogastric tube or gastric tube feeding only	5

B

Updated Swallow Domain Scoring Methodology	Score
Normal, no dysphagia	0
Cough while eating	1
Intermittent dysphagia	2
Dysphagia	3
Nasogastric tube or gastric tube for supplemental feeding	4
Nasogastric tube or gastric tube feeding only	5

Scores clearly delineated
- Each step-wise increase in swallow dysfunction matched with numeric point increase in score

Guideline rescored Swallow domain:

- Normal swallow: appropriate for age (score 0)
- Cough while eating (score 1)
  - Patient eats/drinks, but coughs more than what it would be expected for a child on their age on an appropriate oral intake
- Intermittent dysphagia (score 2)
  - Patient coughs often but not always, when drinking, particularly clear fluids and may cough or choke on certain foods
  - Small sips, straw can help
  - Patient can manage with appropriate size, or avoiding foods not easily swallowed
- Dysphagia (consistent) (score 3)
  - Every time patient drinks, they cough and splutter, particularly clear fluids and the patient struggles to swallow anything solid
  - Liquid may come back down the nose
  - Patient chokes often
  - Food needs to be finely chopped or pureed
- Nasogastric tube or gastric tube for supplemental feeding (score 4)
  - Patient can ingest thickened fluids or pureed food but extra liquids and/or medications need to be given by nasogastric tube
  - It takes very long to eat
  - Sufficient calorie intake cannot be achieved without supplemental feeding by tube
- Nasogastric tube or gastric tube feeding only (score 5)
  - All food and liquids are giving in a tube
  - Oral intake linked to high risk of aspiration

Table S2. Population background data of the qualitative study involving clinical experts and swallow experts

	Total (N=12)	Clinical specialty		
		NPC clinical experts (n=4)	NPC-002 clinical experts (n=4)	Swallow experts (n=4)
**Area of clinical expertise**				
Child neurologist	2 (16.7%)	1 (25.0%)	1 (25.0%)	0
Clinical or biochemical genetics	1 (8.3%)	1 (25.0%)	0	0
Neurologist	0	0	0	0
Speech language, swallowing pathologist	2 (16.7%)	0	0	2 (50.0%)
Speech therapist	1 (8.3%)	0	0	1 (25.0%)
Other[a]	6 (50.0%)	2 (50.0%)	3 (75.0%)	1 (25.0%)
n missing	0	0	0	0
**NPC patients seen per typical year**				
n	12	4	4	4
Mean (SD)	18.2 (14.60)	19.3 (15.09)	9.0 (5.48)	26.3 (17.97)
Median	15.0	16.5	9.0	22.5
Q1, Q3	8.0,24.0	9.5,29.0	4.5,13.5	12.5,40.0
Min, Max	3,50	4,40	3,15	10,50
n missing	0	0	0	0
**NPC patients seen over career**				
n	12	4	4	4
Mean (SD)	65.2 (108.74)	51.5 (38.41)	20.3 (15.50)	123.8 (184.72)
Median	35.0	50.0	17.5	40.0
Q1, Q3	12.5,50.0	28.0,75.0	8.0,32.5	22.5,225.0
Min, Max	6,400	6,100	6,40	15,400
n missing	0	0	0	0
**Typical age of NPC patients (in clinical practice)**				
1 year to 30 years	1 (8.3%)	0	1 (25.0%)	0
2 months to 38 years	1 (8.3%)	1 (25.0%)	0	0
2 months to in their 30s	1 (8.3%)	1 (25.0%)	0	0
5 years to in their 30s	1 (8.3%)	0	0	1 (25.0%)
6 months to 35 years	1 (8.3%)	0	1 (25.0%)	0
6 weeks to 18 years	1 (8.3%)	0	1 (25.0%)	0
Birth to 1 year	1 (8.3%)	0	1 (25.0%)	0
Birth to 17 years	1 (8.3%)	0	0	1 (25.0%)
Birth to 18 years	1 (8.3%)	0	0	1 (25.0%)
Birth to 35 years	1 (8.3%)	1 (25.0%)	0	0
Birth to 72 years	1 (8.3%)	1 (25.0%)	0	0
Birth to 90 years	1 (8.3%)	0	0	1 (25.0%)
**Years treating NPC patients**				
n	8	4	4	n/a
Mean (SD)	16.0 (8.80)	22.3 (7.18)	9.8 (4.99)	n/a
Median	14.0	23.0	9.5	n/a
Q1, Q3	9.5,23.0	17.0,27.5	5.5,14.0	n/a
Min, Max	5,30	13,30	5,15	n/a
n missing	0	0	0	n/a

	Total (N=12)	Clinical specialty		
		NPC clinical experts (n=4)	NPC-002 clinical experts (n=4)	Swallow experts (n=4)
**Years as swallowing specialist**				
n	4	n/a	n/a	4
Mean (SD)	18.8 (10.31)	n/a	n/a	18.8 (10.31)
Median	17.5	n/a	n/a	17.5
Q1, Q3	10.0,27.5	n/a	n/a	10.0,27.5
Min, Max	10,30	n/a	n/a	10,30
n missing	0	n/a	n/a	0
**Currently involved in NPC-002 trial**				
Yes	4 (33.3%)	0	4 (100.0%)	0
No	8 (66.7%)	4 (100.0%)	0	4 (100.0%)
n missing	0	0	0	0
**Previously involved in NPC-002 trial**				
Yes	4 (100.0%)	n/a	4 (100.0%)	n/a
No	0	n/a	0	n/a
n missing	0	n/a	0	n/a
**NPC patients seen in NPC-002 trial**				
n	4	n/a	4	n/a
Mean (SD)	5.8 (2.75)	n/a	5.8 (2.75)	n/a
Median	5.5	n/a	5.5	n/a
Q1, Q3	3.5,8.0	n/a	3.5,8.0	n/a
Min, Max	3,9	n/a	3,9	n/a
n missing	0	n/a	0	n/a
**Patients with neurodegenerative or neurological disease seen per typical year**				
n	4	n/a	n/a	4
Mean (SD)	173.8 (98.77)	n/a	n/a	173.8 (98.77)
Median	160.0	n/a	n/a	160.0
Q1, Q3	97.5,250.0	n/a	n/a	97.5,250.0
Min, Max	75,300	n/a	n/a	75,300
n missing	0	n/a	n/a	0
**Patients with neurodegenerative or neurological disease seen over career**				
n	4	n/a	n/a	4
Mean (SD)	605.0 (461.99)	n/a	n/a	605.0 (461.99)
Median	650.0	n/a	n/a	650.0
Q1, Q3	210.0,1000.0	n/a	n/a	210.0,1000.0
Min, Max	120,1000	n/a	n/a	120,1000
n missing	0	n/a	n/a	0
**Do you have experience assessing swallow dysfunction?**				
Yes	8 (100.0%)	4 (100.0%)	4 (100.0%)	n/a
No	0	0	0	n/a
n missing	0	0	0	n/a

	Total (N=12)	Clinical specialty		
		NPC clinical experts (n=4)	NPC-002 clinical experts (n=4)	Swallow experts (n=4)
**Do you involve a swallow expert to perform functional test of swallow dysfunction?**				
Yes	7 (87.5%)	3 (75.0%)	4 (100.0%)	n/a
No	1 (12.5%)	1 (25.0%)	0	n/a
n missing	0	0	0	n/a

5DNPCCSS: 5-domain Niemann-Pick disease type C Clinical Severity Scale; max: maximum; min: minimum; NPC: Niemann-Pick disease Type C; NPCCSS: Niemann-Pick disease type C Clinical Severity Scale; number of subjects with non-missing data used as the denominator, Q1: 25th percentile, Q3: 75th percentile;,SD: standard deviation

[a]Other areas of clinical expertise included: metabolic pediatrics, pediatric lysosomal storage disorders/NPC, and pediatric gastroenterology

Table S3. Experience of clinical experts and swallow experts involved in the qualitative study with the 5DNPCCSS/NPCCSS

Experience using 5DNPCCSS/NPCCSS	Total (N=8)	Clinical specialty		
		NPC clinical experts (n=4)	NPC-002 clinical experts (n=4)	Swallow experts (n=4)
**Experience using measure**				
Yes	(50.0%)	4 (100.0%)	n/a 0	0
No	4 (50.0%)	0	n/a	4 (100.0%)
n missing	0	0	n/a	0
**Years using measure**				
n	4	4	n/a	n/a
Mean (SD)	5.8 (2.99)	5.8 (2.99)	n/a	n/a
Median	5.0	5.0	n/a	n/a
Q1, Q3	4.0,7.5	4.0,7.5	n/a	n/a
Min, Max	3,10	3,10	n/a	n/a
n missing	0	0	n/a	n/a
**Types of setting used measure**				
Clinical practice	4 (100.0%)	4 (100.0%)	n/a	n/a
Clinical trial*	4 (100.0%)	4 (100.0%)	n/a	n/a
n missing	0	0	n/a	n/a
**Time since last used measure (weeks)**				
n	4	4	n/a	n/a
Mean (SD)	2.5 (2.38)	2.5 (2.38)	n/a	n/a
Median	1.5	1.5	n/a	n/a
Q1, Q3	1.0,4.0	1.0,4.0	n/a	n/a
Min, Max	1,6	1,6	n/a	n/a
n missing	0	0	n/a	n/a
**Do you use a guidance document to score swallow function?**				
Yes	3 (75.0%)	3 (75.0%)	n/a	n/a
No	1 (25.0%)	1 (25.0%)	n/a	n/a
n missing 0 0 n/a n/a	0	0	n/a	n/a

5DNPCCSS: 5-domain Niemann-Pick disease type C Clinical Severity Scale; max: maximum; min: minimum; NPC: Niemann-Pick disease type C; NPCCSS: Niemann-Pick disease type C Clinical Severity Scale; number of subjects with non-missing data used as the denominator, Q1: 25th percentile, Q3: 75th percentile; SD: standard deviation

*Excludes NPC-002 study

Figure S1. Severity of individual R4DNPCCSS domains versus global severity of disease assessed by CGI-S (top) and NPC-cdb (bottom) (NPC-002, FAS)

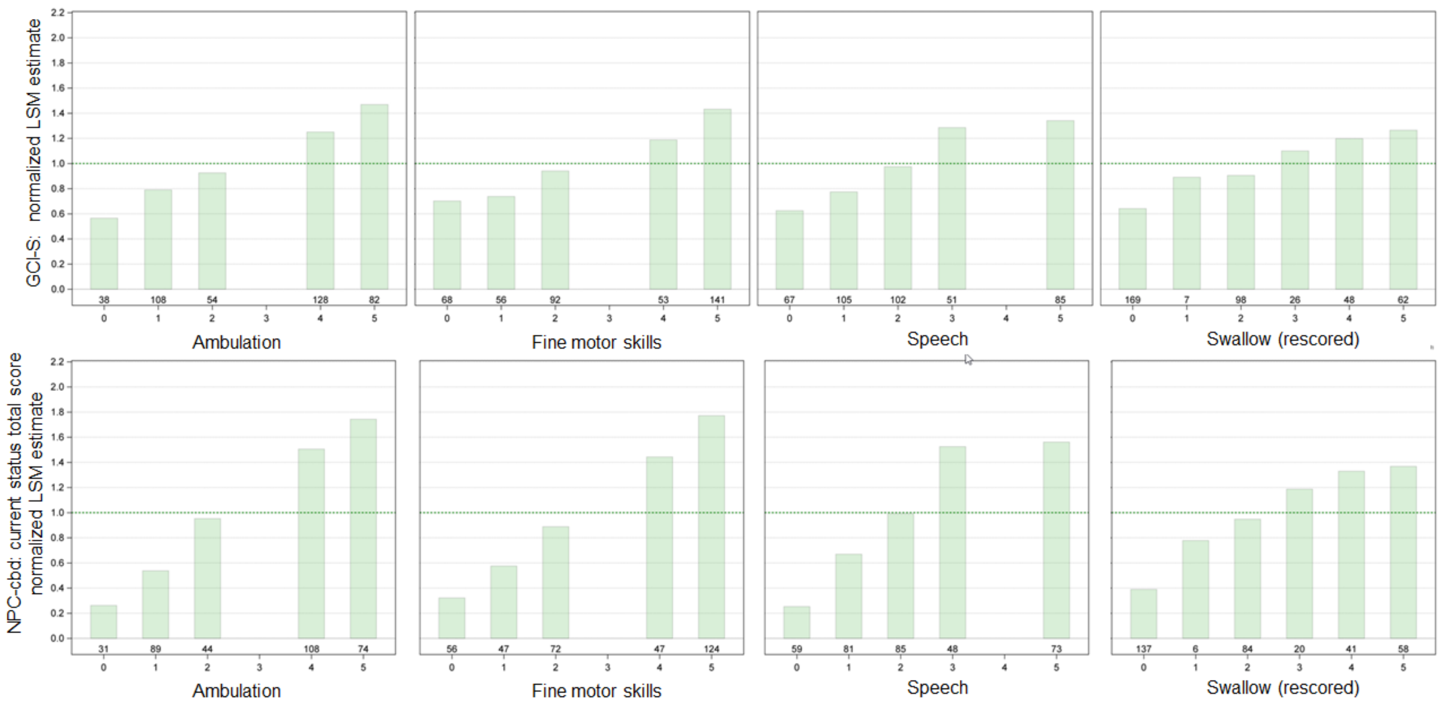

CGI-S: Clinical Global Impression of disease Severity; FAS: full analysis set; LSM: least square mean; NPC-cdb: NPC Clinical Database; R4DNPCCSS: rescored 4-domain Niemann-Pick disease type C Clinical Severity Scale. N = 50 patients. The value of 3 is not an option for rating NPCCSS Ambulation and Fine motor skills, and the value of 4 is not an option for rating NPCCSS Speech. The Swallow scores are the rescored values. Calculation of LS means estimates of the selected efficacy parameters for given outcomes of the R4DNPCCSS domains, using a mixed model with repeated measurements (MMRM) with visit and the relevant NPCCSS domain scores as factors. All data from scheduled visits up to month 60 have been included, ignoring treatment. Only data for patients and visits with data points for both scales are included. To enable comparison across endpoints, CGI-S and NPC-cdb were normalized by dividing with the mean value of the contributing values for each score, meaning that values below 1 are below the mean observed for the entire dataset. Numbers below each of the bars signify the number of contributing observations for that score.

Table S4. 5DNPCCSS and R4DNPCCSS scores at baseline and 12 months of treatment – while on treatment

	N	Baseline	N	12 months
Mean (SD) 5DNPCCSS score				
Arimoclomol	34	12.1 (6.9)	34	13.2 (8.1)
Placebo	16	9.4 (6.4)	15[a]	11.5 (7.7)
Mean (SD) R4DNPCCSS score				
Arimoclomol	34	9.2 (5.8)	34	9.9 (6.7)
Placebo	16	6.7 (5.2)	15[a]	8.7 (6.5)

5DNPCCSS: 5-domain Niemann-Pick disease type C Clinical Severity Scale; R4DNPCCSS: rescored 4-domain Niemann-Pick disease type C Clinical Severity Scale; SD: standard deviation.
[a]One patient in the placebo group had no post-baseline value
